# Harnessing Data Warehousing for Precision in Off-Label Prescription Detection in Psychiatry (PSYHAMM): Retrospective Proof-of-Concept Evaluation Study

**DOI:** 10.2196/71516

**Published:** 2026-09-29

**Authors:** Emmanuel Chevallier, Catherine Letord, Jean Charlet, Marie-Odile Krebs, Emmanuelle Advenier-Iakovlev, Stefan J Darmoni, Julien Grosjean, Romain Leguillon

**Affiliations:** 1 Groupe Hospitalier Universitaire Paris Psychiatrie & Neurosciences Paris France; 2 Department of Biomedical Informatics Centre Hospitalier Universitaire de Rouen Rouen France; 3 Laboratoire d'Informatique Médicale et d'Ingénierie des Connaissances en e-Santé INSERM, Université Paris 13 Sorbonne Université Paris France; 4 Direction de la Recherche Clinique et de l'Innovation Assistance Publique-Hôpitaux de Paris Paris France; 5 Department of Pharmacy Centre Hospitalier Universitaire de Rouen Rouen France

**Keywords:** data warehousing, drugs, information retrieval, off-label, psychiatry

## Abstract

**Background:**

Off-label drug prescribing is prevalent across medicine, including psychiatry, often due to unmet therapeutic needs and inadequate responses to standard treatments. The PSYHAMM (Psychotropes Hors Autorisation de Mise sur le Marché) project, funded by the French Research Agency, investigates these practices. To support this research, a clinical data warehouse (CDW) with advanced data analysis tools was developed and deployed. This system integrates both structured and unstructured data from electronic health records, facilitating comprehensive data analysis. The goal is to improve understanding, regulation, and safety of off-label drug use in psychiatry by providing insights into prescribing patterns and their impacts, ultimately contributing to better clinical guidelines and patient care.

**Objective:**

This study aimed to evaluate the precision (positive predictive value) of a CDW in identifying candidate off-label prescriptions in psychiatry among the cases automatically flagged by the system, rather than its overall accuracy, sensitivity, or specificity.

**Methods:**

The PSYHAMM data analysis involved a retrospective study of pathology-medication pairs to evaluate the precision of a computerized system among system-flagged cases. This system was compared with manual checks performed by a psychiatrist. The evaluation process included verifying if the condition identified by PSYHAMM was documented in the medical record, assessing diagnostic agreement with tolerance for schizoaffective disorders, and ensuring the identified treatment was current or prescribed in the past. Precision was measured as the number of relevant documents retrieved divided by the total number of documents proposed and was computed for the precise diagnosis, the broad diagnosis, and the identified treatment among the flagged cases.

**Results:**

The study analyzed 197 records, identifying 14 unique drug-pathology combinations. Bipolar disorder treated with sodium valproate represented the most cases (108/197, 54.8%), followed by schizophrenia treated with sodium valproate (37/197, 18.8%). The overall precision for detecting off-label situations was 51.3% (101/197). The precise diagnosis achieved a precision of 75.6% (149/197), while the broad diagnosis showed a higher precision of 84.8% (167/197). The identified treatment had a precision of 61.4% (121/197). The primary challenge was temporal discrepancies, such as distinguishing between acute and chronic conditions, which accounted for most of the 48.7% (96/197) of cases that were incorrectly classified.

**Conclusions:**

As a single-center, proof-of-concept evaluation, the PSYHAMM project demonstrates the potential of automated systems to support the identification of off-label prescriptions in psychiatry as a sensitive prescreening step requiring expert validation. The relatively high false-positive rate was driven mainly by temporal discrepancies (drugs prescribed before the index stay, discontinued during the stay, or only hypothetically mentioned) rather than by semantic errors. Future research should focus on integrating real-time data analytics and expanding to multiple institutions to improve the utility of off-label detection systems.

## Introduction

Prescribing off-label drugs remains a prevalent practice in medicine [1,2], extending to critical areas such as the treatment of COVID-19 [3,4]. This approach is particularly common in managing rare diseases, where approved treatments are often limited or nonexistent [5]. Recognizing the importance of understanding and regulating off-label drug use, several institutions have collaborated since 2018. The Laboratory of Medical Informatics and Knowledge Engineering in eHealth, Sainte-Anne Hospital in Paris, and the Rouen University Hospital Department of Digital Health (RUH DDH) have been jointly working on the PSYHAMM (Psychotropes Hors Autorisation de Mise sur le Marché) project, funded by a grant from the French Research Agency. This project aims to investigate off-label drug prescriptions specifically in psychiatry.

Off-label prescribing in psychiatry is widespread, despite frequently lacking robust scientific evidence to support its efficacy [6-9]. This practice can increase the risk of adverse events [10], underscoring the need for thorough evaluation and regulation. Research has shown that off-label prescriptions are often driven by unmet therapeutic needs or the failure of standard treatments. In psychiatry, this can involve prescribing drugs for conditions outside their approved indications, such as using prazosin for posttraumatic stress disorder (PTSD) or valproic acid for borderline personality disorder. Additionally, age-inappropriate prescriptions, such as aripiprazole for behavioral disorders in children, and deviations in dosage or administration methods, such as using supratherapeutic doses of quetiapine for treatment-resistant depression, are common [11,12].

The need for clinical studies to evaluate the benefits and risks of these off-label prescriptions, particularly in psychiatry, is critical. Off-label use often arises from unmet therapeutic needs or extrapolations from on-label uses, sometimes seeking solutions for challenging clinical cases where conventional treatments have failed. For instance, baclofen is sometimes used off-label for alcohol addiction and selegiline for refractory depressive disorder [13,14]. The lack of robust evidence can lead to potential risks, including adverse drug reactions, increased health care costs, and ethical concerns about patient consent and transparency [15-17].

In France, the National Agency for the Safety of Medicines and Health Products offers temporary guidelines for off-label drug use in cases of significant therapeutic need, where the benefit to risk ratio appears favorable based on current scientific evidence. However, much of the prescribing practice in psychiatry does not adhere to these guidelines. Consequently, national and provincial health agencies are increasingly seeking comprehensive lists of off-label prescriptions from private and public hospitals. To address this, the PSYHAMM team has developed a specialized database for off-label drug use, initially focusing on psychiatry and subsequently expanding to other medical fields [18]. This database ensures a standardized format for exporting data, using reference terminologies such as MeSH, NCIt (National Cancer Institute Thesaurus), MedDRA (Medical Dictionary for Regulatory Activities), and SNOMED CT (Systematized Nomenclature of Medicine Clinical Terms), chosen primarily for their free accessibility despite licensing constraints on some terminologies [19].

The RUH DDH has developed an in-house clinical data warehouse (CDW) [20,21] called EDSaN [22] that includes semantic features and several modules, including Doc’EDS [23], a prescreening tool to search patient profiles and identify cohorts in a document-oriented database. EDSaN is a multilevel search engine combining structured and unstructured data. In a CDW, structured data comprise information such as diagnosis-related groups or laboratory results, while unstructured data are derived from various health documents contained in the electronic health record (EHR) and replicated in the CDW, such as discharge summaries, pathology reports, imaging reports, and admission letters. Both diagnoses and treatments were identified using the EDSaN search engine based on keywords (Apache Lucene index; Apache Software Foundation); fuzzy search and advanced natural language processing algorithms are used to detect negations, hypotheses, or family history information [22]. Boolean operators combined with an equation syntax are able to manage keyword combinations. Temporal relationships are not formally detected except for future events. This is a limit of the tool and thus of this study because treatments can occur before the diagnosis for various reasons.

To effectively carry out the PSYHAMM project, these advanced computational tools and systems were deployed at Sainte-Anne Hospital. These tools were essential in integrating and analyzing the vast amounts of data required for the project. The deployment of EDSaN at Sainte-Anne Hospital enabled seamless access to both structured and unstructured data, facilitating comprehensive data analysis. The semantic capabilities of EDSaN allowed for sophisticated data querying and retrieval, ensuring that all relevant information regarding off-label drug prescriptions could be accurately and efficiently identified. Furthermore, the integration of Doc’EDS provided a powerful prescreening mechanism to search patient profiles and identify specific cohorts within the hospital’s database. This functionality was crucial for pinpointing cases of off-label drug use and understanding their context within the broader scope of psychiatric treatment. The deployment of these tools represented a significant technological advancement for Sainte-Anne Hospital, enhancing its ability to support complex research projects such as PSYHAMM (Figure 1).

The aim of this study is to evaluate the precision (positive predictive value) of this CDW in identifying candidate off-label prescriptions in psychiatry among system-flagged cases, using the diverse health documents available at Sainte-Anne Hospital. As only system-flagged records were manually reviewed, the study characterizes precision within this prescreening step and does not estimate the system’s overall accuracy, sensitivity, or specificity. By examining these practices, the PSYHAMM project aims to provide a clearer understanding of off-label drug use in psychiatry, contributing to better clinical guidelines and improved patient safety, and ensuring that they are based on solid evidence and conducted with the highest standards of patient care.

**Figure 1:**
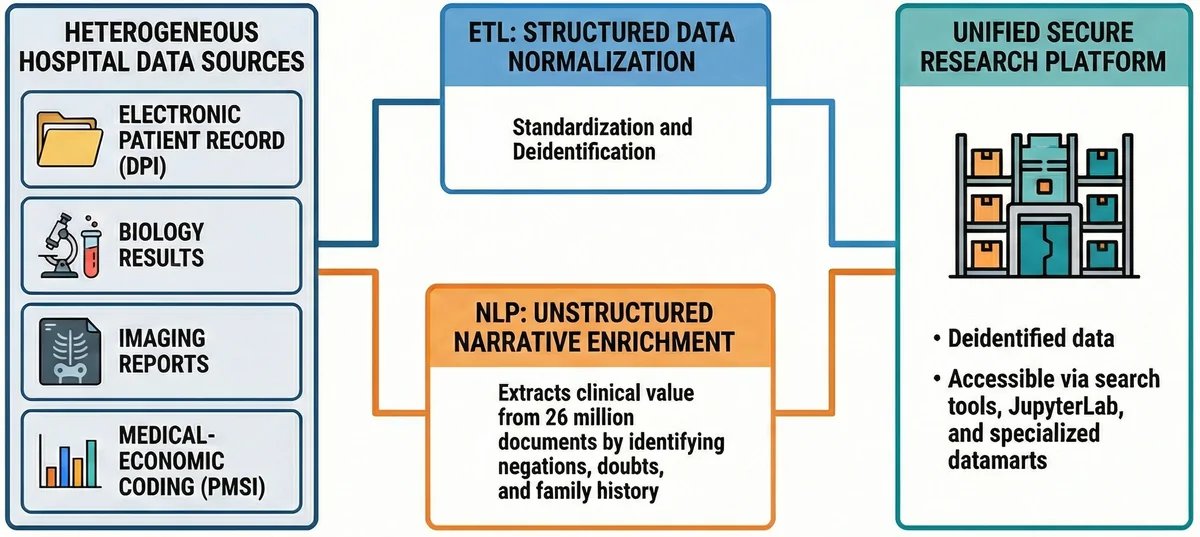
The EDSaN clinical data warehouse integrates data from the hospital information systems using dedicated extract, transform, load (ETL) programs that deidentify, transform, and structure data. Unstructured documents are processed by natural language processing (NLP) techniques to deidentify and detect negations, doubts, and family history content. The final platform integrates dedicated tools to search and process data in a secured environment. DPI: Dossier Patient Informatisé (computerized patient record); PMSI: Programme de Médicalisation des Systèmes d'Information (French hospital discharge database).

## Methods

### Data Analysis and Evaluation

The analysis of PSYHAMM data aims to evaluate the precision of a computerized system in identifying, among system-flagged cases, pathologies and associated off-label treatments. This evaluation is based on comparisons with manual checks performed by a psychiatrist (EC) using a structured evaluation grid based on 4 predefined binary criteria applied systematically to each record: (1) whether the diagnosis identified by PSYHAMM was documented in the medical record (excluding negated, hypothetical, or family history mentions); (2) whether the diagnosis was accurate at the precise or broad level (with a predefined tolerance for schizoaffective disorders being classified as either schizophrenia or bipolar disorder); (3) whether the identified treatment was current or had been prescribed during the relevant period; and (4) whether the drug-pathology pair constituted a confirmed off-label situation, with no other accepted indication present. Borderline or ambiguous cases, particularly those involving atypical diagnostic categorizations, were discussed with a senior psychiatrist (MOK) to establish consistent decision rules for the main ambiguities encountered. Because this reference standard relied on a single primary rater (EC) rather than independent, blinded double coding, no interrater agreement statistic could be computed.

### Retrospective Analysis

A retrospective analysis of pathology-medication pairs was conducted to evaluate precision among system-flagged cases in a clinical context. The data were extracted from a detailed file containing information on diagnoses, administered treatments, and off-label situations. The main variables studied by the expert included the precise diagnosis, which is the exact diagnosis identified by PSYHAMM, and the broad diagnosis. Other variables included medication treatment, referring to the specific treatment prescribed, and off-label detection, which pertains to the identification of off-label prescriptions.

A sample of the database, which automatically detected off-label prescription situations, was extracted to identify unique pathology-medication pairs and calculate their frequency. Precision was measured for the 4 evaluation variables: precise diagnosis, broad diagnosis, medication treatment, and off-label detection. Precision was defined as the number of relevant documents retrieved divided by the total number of documents proposed for a given query, calculated as follows:

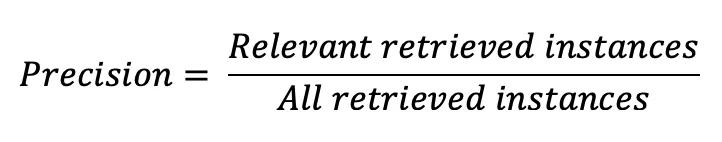



Because the manual evaluation was applied only to the drug-pathology pairs that the system had automatically flagged as potentially off-label, the reference set contained no system-negative cases. Recall (sensitivity), specificity, and the *F*_1_-score could therefore not be estimated, and precision (positive predictive value) was adopted as the primary performance metric. This is consistent with the intended use of the tool as a high-sensitivity prescreening filter followed by expert validation, rather than as a standalone diagnostic classifier.

The relative contribution of each parameter (precise diagnosis, broad diagnosis, and identified treatment) to confirmed off-label status is reported descriptively, using precision values. No multivariable model was fitted because these parameters are themselves components of the manual off-label determination, so such a model would be partly circular and could overstate their apparent predictive value.

### Ethical Considerations

This study was conducted in accordance with the principles of the Declaration of Helsinki. It consisted of a retrospective, secondary analysis of routinely collected, deidentified clinical data extracted from the EDSaN CDW, without any intervention or direct contact with patients. The study was reviewed and approved by the ethics committee of GHU Paris Psychiatrie et Neurosciences (Sainte-Anne). Because the study was classified as research not involving human participants (*recherche n’impliquant pas la personne humaine*) and used exclusively preexisting deidentified records for secondary purposes, individual informed consent was not required. Patients managed at GHU Paris Psychiatrie et Neurosciences (Sainte-Anne) were informed, through institutional information notices, of the possible reuse of their data for research and of their right to object, in accordance with the nonopposition (opt-out) regime applicable to research not involving human participants. All data were deidentified prior to analysis by the automated extract-transform-load pipeline of the EDSaN warehouse, which removes direct identifiers and applies natural language processing to suppress identifying information in free-text documents. Data were stored and analyzed within a secured, access-controlled environment accessible only to authorized members of the research team, and no identifiable patient data are reported in this manuscript.

## Results

### Drug-Pathology Combinations

The 14 drug-pathology combinations identified among the sample of 197 records are presented in Table 1. Bipolar disorder treated with sodium valproate represented the majority of cases, with 108 (54.8%) occurrences, followed by schizophrenia treated with sodium valproate, with 37 (18.8%) occurrences. Other notable combinations include obsessive-compulsive disorder treated with venlafaxine, with 11 (5.6%) occurrences, and attention-deficit/hyperactivity disorder in adults treated with methylphenidate, with 9 (4.6%) occurrences. Each of the remaining combinations accounted for less than 4% of the cases (6/197 or fewer).

**Table 1:** Drug-pathology combinations (N=197)^a^.

Diagnoses	Treatment	Occurrences, n (%)
Bipolar disorder	Sodium valproate	108 (54.8)
Schizophrenia	Sodium valproate	37 (18.8)
Obsessive-compulsive disorder	Venlafaxine	11 (5.6)
Attention-deficit/hyperactivity disorder in adults	Methylphenidate	9 (4.6)
Bipolar disorder	Pramipexole	6 (3.0)
Mood disorders	Topiramate	6 (3.0)
Posttraumatic stress disorder	Venlafaxine	6 (3.0)
Major depressive disorder	Pramipexole	4 (2.0)
Weight loss	Topiramate	3 (1.5)
Hyperphagia	Topiramate	3 (1.5)
Narcolepsy	Methylphenidate	1 (0.5)
Neuralgia	Topiramate	1 (0.5)
Fibromyalgia	Pramipexole	1 (0.5)
Autism spectrum disorder	Methylphenidate	1 (0.5)

^a^Percentages are calculated on the total of 197 system-flagged drug-pathology pairs (N=197).

### Precision of the Diagnostic Parameters

The precision of the diagnostic parameters among system-flagged cases (N=197) is presented in Table 2. The overall precision for confirmed off-label situations was 51.3% (n=101). Precision was 75.6% (n=149) for the precise diagnosis, 84.8% (n=167) for the broad diagnosis, and 61.4% (n=121) for the identified treatment.

**Table 2:** Precision of diagnostic parameters for off-label situations (N=197)^a^.

Parameter	Precision, n (%)
Off-label situation	101 (51.3)
Precise diagnosis	149 (75.6)
Broad diagnosis	167 (84.8)
Identified treatment	121 (61.4)

^a^N=197 system-flagged drug-pathology pairs. Precision is the positive predictive value among flagged cases.

### Analysis of False Positives

Among the 197 drug-pathology pairs studied, 101 (51.3%) were evaluated by the expert as true positives, meaning they were indeed off-label prescriptions (Figure 2). Conversely, 96 (48.7%) pairs were incorrectly identified by the system as off-label (Figure 2). Among these 96 cases, in 76 (79.2%) cases, the patient was not exposed to the drug involved in the off-label prescription. The reasons for this nonexposure are as follows: in 50 (65.8%) cases, it was a prescription prior to the concerned stay, indicated as a history of exposure; in 23 (30.3%) cases, there was a treatment discontinuation during the stay; and in 3 (3.9%) cases, the treatment was mentioned in the report only as a therapeutic possibility (hypothetical; Figure 2). Finally, in 20 (20.8%) of the 96 cases, the diagnosis was incorrect, meaning another diagnosis should have been considered for the studied drug. The results are summarized in Figure 2.

**Figure 2:**
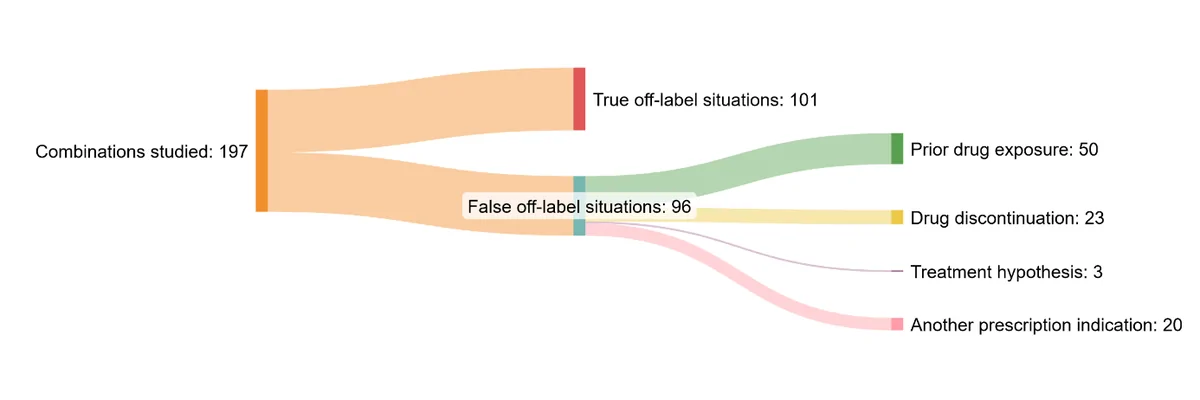
Distribution of drug-pathology combinations and reasons for false-positive off-label detection, in a flow diagram of true and false positives.

The evaluation of diagnoses and treatments, detailing the precision of precise and broad diagnoses, identified treatments, and off-label situations for various pathology-medication pairs, is presented in Table 3. Bipolar disorder treated with sodium valproate showed the highest precision in both precise (90/108, 83.3%) and broad diagnoses (104/108, 96.3%). Schizophrenia treated with sodium valproate had a high treatment identification rate (25/37, 67.6%) but lower off-label situation precision (18/37, 48.6%). Overall, the precision varied substantially across different pathology-medication pairs, highlighting areas for improvement in precise diagnosis and off-label detection (Table 3).

**Table 3:** Evaluation of diagnoses and treatments: precision, identification, and off-label situations^a^.

Diagnoses	Treatments	Occurrences, n (%)	Precise diagnosis, n (%)	Broad diagnosis, n (%)	Identified treatment, n (%)	Off-label situation, n (%)
Bipolar disorder	Sodium valproate	108 (54.8)	90 (83.3)	104 (96.3)	68 (63.0)	65 (60.2)
Schizophrenia	Sodium valproate	37 (18.8)	28 (75.7)	30 (81.1)	25 (67.6)	18 (48.6)
Obsessive-compulsive disorder	Venlafaxine	11 (5.6)	8 (72.7)	8 (72.7)	7 (63.6)	5 (45.5)
Attention-deficit/hyperactivity disorder in adults	Methylphenidate	9 (4.6)	8 (88.9)	8 (88.9)	5 (55.6)	5 (55.6)
Bipolar disorder	Pramipexole	6 (3.0)	4 (66.7)	4 (66.7)	4 (66.7)	2 (33.3)
Mood disorders	Topiramate	6 (3.0)	1 (16.7)	3 (50.0)	4 (66.7)	3 (50.0)
Posttraumatic stress disorder	Venlafaxine	6 (3.0)	3 (50.0)	3 (50.0)	5 (83.3)	2 (33.3)
Major depressive disorder	Pramipexole	4 (2.0)	3 (75.0)	3 (75.0)	2 (50.0)	1 (25.0)
Weight loss	Topiramate	3 (1.5)	0 (0.0)	0 (0.0)	0 (0.0)	0 (0.0)
Hyperphagia	Topiramate	3 (1.5)	2 (66.7)	2 (66.7)	0 (0.0)	0 (0.0)
Narcolepsy	Methylphenidate	1 (0.5)	0 (0.0)	0 (0.0)	0 (0.0)	0 (0.0)
Neuralgia	Topiramate	1 (0.5)	1 (100.0)	1 (100.0)	0 (0.0)	0 (0.0)
Fibromyalgia	Pramipexole	1 (0.5)	1 (100.0)	1 (100.0)	0 (0.0)	0 (0.0)
Autism spectrum disorder	Methylphenidate	1 (0.5)	0 (0.0)	0 (0.0)	1 (100.0)	0 (0.0)

^a^N=197 system-flagged drug-pathology pairs; n (%) values are calculated within each drug-pathology pair.

## Discussion

### Principal Results

#### Innovative Method for Analyzing Off-Label Prescriptions

The PSYHAMM project represents an innovative approach to analyzing off-label drug prescriptions, leveraging advanced computational tools and CDWs to enhance detection precision. Traditional methods of identifying off-label use often rely on manual chart reviews and self-reporting, which are time consuming and prone to human error. By contrast, the PSYHAMM system uses a computerized algorithm that scans EHRs for pathology-treatment pairs, enabling a more efficient and scalable solution. One of the key components of this method is the use of semantic technologies and multilevel search engines, such as EDSaN. These tools integrate structured and unstructured data from various health documents, including discharge summaries, pathology reports, imaging reports, and admission letters. The ability to process both types of data enhances the system’s capacity to detect nuanced and complex off-label prescribing patterns that might otherwise be overlooked [24-26].

Moreover, the inclusion of prescreening tools such as Doc’EDS allows for the identification of patient cohorts and the precise querying of patient profiles. This capability is particularly valuable in psychiatry, where the range of potential off-label uses is broad and often involves medications initially approved for entirely different conditions. For instance, the off-label use of antipsychotics in treating conditions such as PTSD or borderline personality disorder has been well-documented, though it frequently lacks robust supporting evidence [11,27].

The integration of these advanced data processing tools with clinical oversight, as seen in the PSYHAMM project, offers a promising model for future studies. By combining automated data extraction and analysis with manual verification by clinical experts, this approach ensures both efficiency and precision, addressing many of the limitations of previous methods.

There are few automated systems specifically dedicated to detecting off-label prescriptions from patient records, particularly in psychiatry. Comparable work relies mainly on the analysis of medical-administrative databases or EHRs to match drugs and diagnoses, such as in the studies by Radley et al [2], Eguale et al [17], or Knez and Žitnik [28], with well-identified methodological limitations. Approaches involving electronic phenotyping and semantic exploration of EHRs, as described by Hripcsak and Albers [29], show significant potential but highlight the difficulties associated with temporality and clinical context. In this perspective, PSYHAMM is part of an automated prescreening approach that combines structured and unstructured data to identify potentially off-label situations requiring clinical validation.

#### Encouraging Results With Automatic Detection

The results of the PSYHAMM project are encouraging, demonstrating the potential of automated systems to accurately identify off-label prescriptions. The overall precision of 51.3% (Table 2) indicates that more than half of the automatically detected off-label prescriptions were confirmed as correct upon manual review. This level of precision is a significant achievement, given the complexity of psychiatric diagnoses and treatments.

The distribution of drug-pathology combinations and the precision of diagnostic parameters are highlighted (Tables 1 and 2). For example, bipolar disorder treated with sodium valproate was the most common combination identified, with a high frequency of 54.8% (Table 1). The precision of specific diagnoses such as this one was robust, reflecting the system’s capability to correctly match clinical documentation with the identified conditions and treatments.

Descriptively, the identified treatment and the broad diagnosis showed good precision, underscoring the importance of accurate treatment identification for the overall precision of off-label prescreening.

However, it is important to note that while the automated system was effective in identifying off-label prescriptions, not all elements were consistently found in the medical records. For example, of the 197 cases analyzed, the system incorrectly identified 96 (48.7%; Figure 2) prescriptions as off-label. This discrepancy highlights the need for continuous refinement of the algorithms and further integration of comprehensive clinical data to enhance precision. The discrepancy observed between the high precision of diagnostic identification and the lower precision of detecting off-label situations can be explained by the complexity of linking diagnosis and treatment. While diagnoses and medications are generally well identified in isolation, correctly associating them requires considering the clinical context and, above all, timing, which is not fully modeled in the tool. The 48.7% false positives therefore do not correspond mainly to semantic errors but to clinically ambiguous situations: previous treatments, discontinued during the hospital stay, or simply mentioned as a therapeutic hypothesis. This high false-positive rate (ie, a modest precision: 51.3%) reflects a deliberate methodological choice, favoring broad and sensitive identification of potentially off-label situations suitable for surveillance or prescreening purposes but requiring human validation before any clinical interpretation or decision-making. In this context, the *F*_1_-score was not relevant. Accordingly, PSYHAMM should be understood as a sensitive prescreening and surveillance instrument that flags candidate situations for mandatory expert review, not as an autonomous classifier of off-label prescribing. In this intended use, a higher false-positive rate is an accepted trade-off for maximizing sensitivity, provided that every flagged case is subsequently validated by a clinician.

#### Temporal Issues and Known Challenges

One of the primary challenges identified in this study is the issue of temporality, specifically the distinction between past or present diagnoses. This problem is not unique to the PSYHAMM project and has been recognized in previous research [29,30]. In many cases, the automated system failed to account for changes in the patient’s condition over time, leading to incorrect classification of prescriptions as off-label.

The temporal dimension of drug prescribing is crucial, particularly in psychiatry where treatment regimens often evolve based on the patient’s response and emerging symptoms. For example, a medication initially prescribed for acute symptoms may become part of a long-term management plan, altering its status from off-label to on-label as more evidence supports its use for chronic conditions.

This study found that a significant portion of the errors were due to the patient not being currently exposed to the medication identified in the off-label prescription (Figure 2). The reasons for these included prescriptions that were prior to the stay, treatment discontinuations during the stay, and medications mentioned as hypothetical treatment options. These findings align with previous research, indicating that the temporal aspect of medication use is a critical factor in accurately identifying off-label prescriptions [17].

Addressing these temporal challenges requires sophisticated data integration and analysis techniques capable of tracking patient history and treatment progression over time. Future iterations of the PSYHAMM system could benefit from incorporating longitudinal data analysis and machine learning models that can predict and adjust for changes in treatment status. In this context, we should also study the potential contribution of large language models to constructing the temporal sequence of patient events [31].

#### Implications for Clinical Practice and Policy

More cautiously, and within the limits of this single-center evaluation, tools such as PSYHAMM may support patient safety by helping clinicians identify candidate off-label prescriptions for expert review, provided that every flagged case is validated before any clinical interpretation. Broader claims about health policy or regulatory monitoring would require multicenter validation and formal performance evaluation and are beyond the scope of the present data.

### Limitations

Despite the promising results, there are several limitations to the current study that must be addressed in future research. One major limitation is the reliance on the accuracy of EHRs, which can vary significantly in terms of completeness and consistency. Incomplete or inaccurate records can lead to false positives or negatives in off-label detection.

Performance was characterized using precision (positive predictive value) alone. Because manual review was restricted to system-flagged candidates, the reported 51.3% precision—and the corresponding 48.7% false-positive rate—quantify only the proportion of flagged candidates that were genuine off-label situations, and not the system’s ability to retrieve all true off-label prescriptions. A complete performance characterization, including recall against an exhaustively annotated reference standard, remains necessary and is planned for future work.

Additionally, the manual verification process, while essential for ensuring precision, is time consuming and subject to human error. A key limitation of the present study is that the reference evaluation relied on a single primary rater (EC), with ambiguous or borderline cases adjudicated jointly with a senior psychiatrist (MOK) according to predefined decision rules. Although this approach provided expert-level judgment and ensured consistent handling of the main ambiguity encountered (the categorization of schizoaffective disorders), it does not constitute an independent, blinded double-coding procedure. Consequently, no formal interrater agreement statistic (eg, Cohen κ) could be computed, and the possibility of single-rater bias cannot be excluded. This is an important constraint on the robustness of the validation; independent double coding with measurement of interrater agreement is a priority for future work and a prerequisite before any deployment for clinical or regulatory use.

Another limitation is the focus on a single institution (Sainte-Anne Hospital), which may limit the generalizability of the findings. Expanding the study to include multiple institutions with diverse patient populations and health care practices could provide a more comprehensive understanding of off-label prescribing patterns and the generalizability of the PSYHAMM approach [32].

A further limitation concerns the size and composition of the evaluation sample. The analysis was based on 197 system-flagged drug-pathology pairs, of which more than half (54.8%) corresponded to a single pair (bipolar disorder treated with sodium valproate) and 73.6% to only 2 valproate-related pairs. This distribution reflects the real-world frequency of off-label prescribing at the study site, where valproate is used for several psychiatric indications, rather than a sampling artifact; nonetheless, it limits the precision and stability of per-pair estimates for less frequent combinations, so results for rare pairs should be regarded as preliminary. As a single-center, proof-of-concept evaluation, the study was designed to assess feasibility rather than to provide population-level prevalence estimates; larger, multicenter samples with a more balanced, prospectively defined sampling strategy will be required to confirm and generalize these findings.

Future research should also investigate the integration of additional data sources, such as pharmacy records, to provide a more complete view of medication use, and the use of longitudinal, temporally aware models better able to handle the timing of diagnosis and treatment.

The work presented here was developed solely at Sainte-Anne Hospital. However, EDSaN has been developed and is routinely used at RUH, which covers numerous medical specialties, suggesting that it could be widely adopted. Nevertheless, RUH does not have a psychiatric department, and this possibility will need to be evaluated.

### Conclusions

The PSYHAMM project demonstrates a promising approach to the automated detection of off-label drug prescriptions in psychiatry. By leveraging advanced computational tools and integrating clinical oversight, the system achieves a significant level of precision in identifying off-label use, although challenges remain in addressing temporal issues and ensuring data completeness. The findings underscore the importance of continuous refinement and expansion of automated systems to enhance their reliability and effectiveness in clinical practice. Future research should focus on integrating additional data sources, expanding to multiple institutions, and incorporating advanced analytics to further improve the precision and utility of off-label detection systems.

## Abbreviations

CDW: clinical data warehouse
EHR: electronic health record
MedDRA: Medical Dictionary for Regulatory Activities
MeSH: Medical Subject Headings
NCIt: National Cancer Institute Thesaurus
PSYHAMM: Psychotropes Hors Autorisation de Mise sur le Marché
PTSD: posttraumatic stress disorder
RUH: Rouen University Hospital
RUH DDH: Rouen University Hospital Department of Digital Health
SNOMED CT: Systematized Nomenclature of Medicine Clinical Terms
